# *Nigella sativa* supplementation to treat symptomatic mild COVID-19: A structured summary of a protocol for a randomised, controlled, clinical trial

**DOI:** 10.1186/s13063-020-04647-x

**Published:** 2020-08-08

**Authors:** Abdulrahman E. Koshak, Emad A. Koshak, Abdullah F. Mobeireek, Mazen A. Badawi, Siraj O. Wali, Husam M. Malibary, Ali F. Atwah, Meshari M. Alhamdan, Reem A. Almalki, Tariq A. Madani

**Affiliations:** 1 Department of Natural Products and Alternative Medicine, Faculty of Pharmacy, King Abdulaziz University, Jeddah, Saudi Arabia; 2 Department of Medicine, Faculty of Medicine, King Abdulaziz University, Jeddah, Saudi Arabia; 3 Department of Medicine, King Faisal Specialist Hospital and Research Centre, Riyadh, Saudi Arabia; 4 Department of Pediatrics, Faculty of Medicine, King Abdulaziz University, Rabigh, Saudi Arabia; 5 University Medical Services Centre, King Abdulaziz University, Jeddah, Saudi Arabia

**Keywords:** COVID-19, SARS-CoV-2, Randomised controlled trial, protocol, *Nigella sativa*, black seed, phytotherapy

## Abstract

**Objectives:**

To investigate the potential efficacy of *Nigella sativa* (NS) oil supplementation on the outcomes of patients with mild Coronavirus Disease 2019 (COVID-19).

**Trial design:**

Prospective, two-arm, parallel-group, randomised (1:1 allocation ratio), open-label, controlled, exploratory phase II clinical trial of oral NS oil in patients with mild COVID-19.

**Participants:**

Inclusion Criteria:

- Patients with mild COVID19 (defined as upper respiratory tract infection symptoms in the absence of clinical or radiological signs of pneumonia).

- Adult (18 - 65 years old).

- Written informed consent by the patient (or legally authorized representative) prior to initiation of any study procedures.

- All patients should understand and agree to comply with planned study procedures.

- Polymerase chain reaction (PCR)-confirmed infection with Severe Acute Respiratory Syndrome Coronavirus-2 (SARS-CoV-2) from throat swab.

Exclusion Criteria:

- Patients with pneumonia or severe illness requiring admission to intensive care unit.

- Severe chronic kidney disease (i.e. estimated glomerular filtration rate [eGFR] < 30 mL/min) or end stage renal disease requiring dialysis

- Severe chronic liver disease (Alanine transaminase [AlT] or Aspartate transaminase [AST] > 5 times the upper limit of normal).

- Pregnancy or breast feeding.

- Anticipated transfer within 72 hours to another hospital that is not a study site.

- Allergy to the study medication

The trial is currently conducted on patients recruited from King Abdulaziz University Hospital, Jeddah, Saudi Arabia.

**Intervention and comparator:**

Intervention group: *Nigella sativa* oil (MARNYS® Cuminmar) 500 mg softgel capsules, one capsule orally twice daily for 10 days plus standard of care treatment (antipyretic, antitussive).

Comparator group: standard of care treatment.

**Main outcomes:**

Proportion of patients who clinically recovered (defined as 3 days of no symptoms) within 14 days after randomisation.

**Randomisation:**

Patients will be randomly assigned to treatment or control groups in a 1:1 ratio using a computer-generated randomization scheme (Random permuted blocks of 10) developed using the web-based program: http://www.randomization.com.

**Blinding (masking):**

No blinding.

**Numbers to be randomised (sample size):**

Up to 200 eligible patients will be randomly assigned to either treatment or control groups.

**Trial Status:**

Protocol version 1, as of July 14, 2020. Recruitment was started on May 21, 2020. The intended completion date is December 31, 2020.

**Trial registration:**

ClinicalTrials.gov Identifier: NCT04401202. Date of trial registration: May 26, 2020.

**Full protocol:**

The full protocol is attached as an additional file, accessible from the Trials website (Additional file 1). In the interest of expediting dissemination of this material, the familiar formatting has been eliminated; this letter serves as a summary of the key elements of the full protocol.

## Supplementary information

**Additional file 1.**

## Data Availability

Only investigators will have access to the final trial dataset.

