# Supplementary material for: Nigella sativa supplementation to treat symptomatic mild COVID-19: A structured summary of a protocol for a randomised, controlled, clinical trial
Source: Trials. 2020 Aug 8;21:703. doi: 10.1186/s13063-020-04647-x (PMC7414256; doi:10.1186/s13063-020-04647-x)
Supplement: Supplementary file 1 — Additional file 1. [file 13063_2020_4647_MOESM1_ESM.doc]

**Clinical Research Protocol**

Effects of *Nigella Sativa* as a treatment of patients with upper respiratory

tract infection caused by SARS-cononavirus-2: a prospective, randomized, open-label, controlled clinical study

| Protocol Number: | NA |
| --- | --- |
| Version Date: | 14.07.2020 |
| Investigational Product: | *Nigella sativa* oil (CUMINMAR) |
| IND Number: | NA |
| Development Phase: | Phase II |
| Sponsor: | Self-sponsored  King Abdulaziz University  Jeddah, Saudi Arabia |
| Funding Organization: | NA |
| Principal Investigator: | Name: Dr Abdulrahman Koshak  E-mail: aekoshak@kau.edu.sa |

**PROTOCOL AGREEMENT**

I have read the protocol specified below. In my formal capacity as Investigator, my duties include ensuring the safety of the study subjects enrolled under my supervision and providing [Dr Abdulrahman Koshak] with complete and timely information, as outlined in the protocol. It is understood that all information pertaining to the study will be held strictly confidential and that this confidentiality requirement applies to all study staff at this site. Furthermore, on behalf of the study staff and myself, I agree to maintain the procedures required to carry out the study in accordance with accepted GCP principles and to abide by the terms of this protocol.

Protocol Number: NA

Protocol Title: Effects of *Nigella Sativa* as a treatment of patients with upper respiratory

tract infection caused by SARS-cononavirus-2: a prospective, randomized, open-label, controlled clinical study

Protocol Date: 14.07.2020

|  | | |  | 14.07.2020 |
| --- | --- | --- | --- | --- |
| *Investigator Signature* | | |  | *Date* |
| Dr Abdulrahman Koshak | | | | |
| *Print Name and Title* | | | | |
| *Site #* | NA |  | | |
| *Site Name* | King Abdulaziz University Hospital | | | |
| *Address* | Jeddah 21589 | | | |
|  | Saudi Arabia | | | |
|  |  | | | |
|  |  | | | |
| *Phone Number* | 012-6408222 | | | |

**TABLE OF CONTENTS**

[1 BACKGROUND 4](#__RefHeading___Toc262733374)

1.1 Overview of Non-Clinical Studies [9](#__RefHeading___Toc262733375)

[1.2 Overview of Clinical Studies 10](#__RefHeading___Toc262733376)

[2 STUDY RATIONALE 11](#__RefHeading___Toc262733377)

[2.1 Risk / Benefit Assessment 11](#__RefHeading___Toc262733378)

[3 STUDY OBJECTIVES 12](#__RefHeading___Toc262733379)

[3.1 Primary Objective 12](#__RefHeading___Toc262733380)

[3.2 Secondary Objectives 12](#__RefHeading___Toc262733381)

[4 STUDY DESIGN 12](#__RefHeading___Toc262733382)

[4.1 Study Overview 12](#__RefHeading___Toc262733383)

[5 Criteria for evaluation 12](#__RefHeading___Toc262733384)

[5.1 Primary Efficacy Endpoint 12](#__RefHeading___Toc262733385)

[5.2 Secondary Efficacy Endpoints 12](#__RefHeading___Toc262733386)

[5.3 Safety Evaluations 13](#__RefHeading___Toc262733387)

[5.4 Other Evaluations (include only if applicable) 13](#__RefHeading___Toc262733388)

[6 SUBJECT SELECTION 13](#__RefHeading___Toc262733389)

[6.1 Study Population 13](#__RefHeading___Toc262733390)

[6.2 Inclusion Criteria 13](#__RefHeading___Toc262733391)

[6.3 Exclusion Criteria 13](#__RefHeading___Toc262733392)

[7 Concurrent Medications 13](#__RefHeading___Toc262733393)

[7.1 Allowed 13](#__RefHeading___Toc262733394)

[7.2 Prohibited 13](#__RefHeading___Toc262733395)

[8 STUDY TREATMENTS 14](#__RefHeading___Toc262733396)

[8.1 Method of Assigning Subjects to Treatment Groups 14](#__RefHeading___Toc262733397)

[8.2 Blinding 14](#__RefHeading___Toc262733398)

[8.3 Test and Control Formulation 14](#__RefHeading___Toc262733399)

[8.4 Supply of Study Medication at the Site 14](#__RefHeading___Toc262733400)

[8.5 Study Medication Accountability 15](#__RefHeading___Toc262733401)

[8.6 Measures of Treatment Compliance 15](#__RefHeading___Toc262733402)

[9 STUDY PROCEDURES AND GUIDELINES 15](#__RefHeading___Toc262733403)

[9.1 Clinical Assessments 15](#__RefHeading___Toc262733404)

[9.2 Clinical Laboratory Measurements (include sections as appropriate) 16](#__RefHeading___Toc262733405)

[9.3 Pharmacokinetic Measurements **Error! Bookmark not defined.**](#__RefHeading___Toc262733406)

[9.4 Research Laboratory Measurements (include sections as appropriate) **Error! Bookmark not defined.**](#__RefHeading___Toc262733407)

[10 EVALUATIONS BY VISIT 16](#__RefHeading___Toc262733408)

[10.1 Visit 1 (Day/Week/Month #) 16](#__RefHeading___Toc262733409)

[10.2 Visit 2 (Day/Week/Month # include visit window) **Error! Bookmark not defined.**](#__RefHeading___Toc262733410)

[10.3 Visit 3 (Day/Week/Month # include visit window) **Error! Bookmark not defined.**](#__RefHeading___Toc262733411)

10.4 Visit 4 (Day/Week/Month # include visit window) [**Error! Bookmark not defined.**](#__RefHeading___Toc262733412)

[10.5 Visit 5 (Follow-up or Day/Week/Month # include visit window) **Error! Bookmark not defined.**](#__RefHeading___Toc262733413)

[10.6 Early Withdrawal Visit **Error! Bookmark not defined.**](#__RefHeading___Toc262733414)

[11 ADVERSE Experience REPORTING AND DOCUMENTATION 16](#__RefHeading___Toc262733415)

[11.1 Adverse Events 16](#__RefHeading___Toc262733416)

[11.2 Serious Adverse Experiences (SAE) 18](#__RefHeading___Toc262733417)

[11.3 Protocol Defined Important Medical Findings Requiring Real Time Reporting **Error! Bookmark not defined.**](#__RefHeading___Toc262733418)

[11.4 Medical Monitoring 18](#__RefHeading___Toc262733419)

[11.5 Safety Management Plan](#__RefHeading___Toc262733420) 4

[12 DISCONTINUATION And Replacement of subjects 18](#__RefHeading___Toc262733421)

[12.1 Withdrawal of Subjects 18](#__RefHeading___Toc262733422)

[12.3 Replacement of Subjects 19](#__RefHeading___Toc262733423)

[13 PRotocol Violations 19](#__RefHeading___Toc262733424)

[14 DATA SAFETY MONITORING (optional Section – Include when appropriate) Error! Bookmark not defined.](#__RefHeading___Toc262733425)

[15 STATISTICAL METHODS AND CONSIDERATIONS 20](#__RefHeading___Toc262733426)

[15.1 Data Sets Analyzed 20](#__RefHeading___Toc262733427)

[15.2 Demographic and Baseline Characteristics 20](#__RefHeading___Toc262733428)

[15.3 Analysis of Primary Endpoint 20](#__RefHeading___Toc262733429)

[15.4 Analysis of Secondary Endpoints 20](#__RefHeading___Toc262733430)

[15.5 Interim Analysis **Error! Bookmark not defined.**](#__RefHeading___Toc262733431)

[15.6 Sample Size and Randomization 20](#__RefHeading___Toc262733432)

[16 DATA COLLECTION, RETENTION AND MONITORING 20](#__RefHeading___Toc262733433)

[16.1 Data Collection Instruments 20](#__RefHeading___Toc262733434)

[16.2 Data Management Procedures 21](#__RefHeading___Toc262733435)

[16.3 Data Quality Control and Reporting 21](#__RefHeading___Toc262733436)

[16.4 Archival of Data 21](#__RefHeading___Toc262733437)

[16.5 Availability and Retention of Investigational Records 21](#__RefHeading___Toc262733438)

[16.6 Monitoring 21](#__RefHeading___Toc262733439)

[16.7 Subject Confidentiality 21](#__RefHeading___Toc262733440)

[17 ADMINISTRATIVE, ETHICAL, REGULATORY CONSIDERATIONS 22](#__RefHeading___Toc262733441)

[17.1 Protocol Amendments 22](#__RefHeading___Toc262733442)

[17.2 Institutional Review Boards and Independent Ethics Committees 22](#__RefHeading___Toc262733443)

[17.3 Informed Consent Form 23](#__RefHeading___Toc262733444)

[17.4 Publications 23](#__RefHeading___Toc262733445)

[17.5 Investigator Responsibilities 23](#__RefHeading___Toc262733446)

List of Abbreviations

***Add all other abbreviations referenced in the protocol and delete any not referenced in the protocol.***

| **NS** | *Nigella sativa* |
| --- | --- |
| **CRF** | case report form |
| **SFDA** | Saudi Food and Drug Authority |
| **ICF** | informed consent form |
| **ICH** | International Conference on Harmonisation |
| **IEC** | Independent Ethics Committee |
| **IRB** | Institutional Review Board |
| **PI** | Principal Investigator |
| **SAE** | serious adverse experience |

Protocol Synopsis

| **TITLE** | Effects of *Nigella Sativa* as a treatment of patients with upper respiratory tract infection caused by SARS-cononavirus-2: a prospective, randomized, open-label, controlled clinical study |
| --- | --- |
|  |  |
| **SPONSOR** | Self-sponsored |
|  |  |
| **FUNDING ORGANIZATION** | NA |
| **NUMBER OF SITES** | 1 |
|  |  |
| **RATIONALE** | The world is currently facing a crisis because of this potentially fatal  COVID-19 outbreak with no proven efficacy yet of any drug therapy, while vaccination is not expected until 2021. Therefore, this study is intended to evaluate the potential efficacy for using *Nigella sativa* (NS) oil in patients with COVID-19. |
|  |  |
| **STUDY DESIGN** | Prospective, two-arm, parallel-group, randomised (1:1 allocation ratio), open-label, controlled, exploratory phase II clinical trial |
|  |  |
| **PRIMARY OBJECTIVE** | The primary objective is to assess the clinical efficacy as measured by the change in clinical recovery period over the 10 days treatment period. |
|  |  |
| **SECONDARY OBJECTIVES** | Assess the effect of NS on COVID-19 symptoms recovery. |
|  |  |
| **NUMBER OF SUBJECTS** | 200 |
|  |  |
| **SUBJECT SELECTION**  **CRITERIA** | Inclusion Criteria:  -Patients with mild COVID19 (defined as upper respiratory tract infection symptoms in the absence of clinical or radiological signs of pneumonia).  -Adult (18 - 65 years old).  -Written informed consent by the patient (or legally authorized representative) prior to initiation of any study procedures.  -All patients should understand and agree to comply with planned study procedures.  -Polymerase chain reaction (PCR)-confirmed infection with Severe Acute Respiratory Syndrome Coronavirus-2 (SARS-CoV-2).  Exclusion Criteria:  -Patients with pneumonia or severe illness requiring admission to intensive care unit.  -Asymptomatic patients.  -Severe chronic kidney disease (i.e. estimated glomerular filtration rate [eGFR] < 30 mL/min) or end stage renal disease requiring dialysis  -Severe chronic liver disease (Alanine transaminase [AlT] or Aspartate transaminase [AST] > 5 times the upper limit of normal).  -Pregnancy or breast feeding.  -Anticipated transfer within 72 hours to another hospital that is not a study site.  -Allergy to the study medication. |
|  |  |
| **TEST PRODUCT, DOSE, AND ROUTE OF ADMINISTRATION** | SFDA-licensed Marnys CUMINMAR *Nigella sativa* oil 500mg softgel capsules in oral twice daily dose for 10 days |
|  |  |
| **CONTROL PRODUCT, DOSE AND ROUTE OF ADMINISTRATION** | *NA* |
|  |  |
| dURATION OF SUBJECT PARTICIPATION AND DURATION OF STUDY | Subjects will be on study for up to 14 days  **Screening:** up to 7 days  **Treatment:** 10 days  **Follow-up:** 14 days  The total duration of the study is expected to be 12 months |
| **E** |  |
| **CONCOMMITANT MEDICATIONS** | Allowed: Any medication with no interaction with NS  Prohibited: ANTICOAGULANT/ANTIPLATELET DRUGS, CNS DEPRESSANTS, CYCLOSPORINE, IMMUNOSUPPRESSANTS |
|  |  |
| **Efficacy Evaluations** | Clinical monitoring for 14 days |
| ***Primary endpoint*** | Proportion of patients who clinically recovered (defined as 3 days of no symptoms) within 14 days after date of onset of symptoms. |
| ***Secondary endpoints*** | - Improvement of Clinical Parameters:   o Time to full clinical recovery  o Time to progression to pneumonia or hypoxia  o Duration of illness  o Discharge to home  o Normalization of chest radiograph.  o Rate of complications: ICU admission, noninvasive ventilator, superadded infection  o Death.   - Normalization of Routine Laboratory Tests:   o Complete Blood Count (CBC)  o Erythrocyte sedimentation rate (ESR)  o C-reactive protein  o D-dimer  o Liver function test  o Renal function test  o Lactate Dehydrogenase (LDH),  o Immunoglobulins (IgA, IgE, IgG, IgM),  o Compliments (c3, c4),  o Chest X-ray, CT scan  o COVID-19 PCR  o Immune phenotype (CD3 T Cell, CD4 T Cell, CD8 T Cell, CD19 B Cell, CD20 B Cell, NK Cell). |
| **Other Evaluations** | Inflammatory cytokines (IL-1β, IL-1Ra, IL-2, IL-6, IL-10, INF-alpha, INF-gamma, Eotaxin, RANTES & TNF-α). |
| **Safety Evaluations** | Incidence of adverse events from the investigational drug or transfer to ICU |
| **Planned Interim Analyses** | When approximately 50% of patients have completed the study, an interim analysis for safety will be conducted by an independent data monitoring committee. Serious adverse events will be monitored by the research team on an ongoing basis throughout the study. |
|  |  |
| **STATISTICS**  **Primary Analysis Plan** | After 10 days of intervention, the Proportion of patients who clinically recovered on day 14 will be compared with control group. Student t test will be used to compare means of both groups. |
| **Rationale for Number of Subjects** | To achieve a power of 80% and a two-tailed p-value of <0.05 of an assumed 40% difference between the proportion of patients with clinical cure among the NS group in comparison with the control group, a total of at least 200 patients assigned randomly into the two arms (ie 100 patients per arm) will be needed to be included in the study as calculated using G*Power version 3.1.9.2 (Heinrich-Heine-University Düsseldorf, Düsseldorf, Germany). |

# BACKGROUND

The clinical disease termed COVID-19 is caused by a novel betacorona virus, now named SARS-CoV-2. The most common symptoms being reported are fever, cough or chest tightness, and dyspnoea. Most cases are reported to experience a mild illness course (1). Thirty-two per cent required intensive care unit (ICU) admission for respiratory support (1). Laboratory features in this case series commonly included leukopenia, lymphopenia and raised aspartate aminotransferase (1). An epidemiological study of 99 cases of 2019 novel coronavirus pneumonia in Wuhan, China. found an overall mortality rate of 11% with 23% of patients requiring ICU admission (1). Currently, the World Health Organization (WHO) upgraded the status of the COVID-19 outbreak from epidemic to pandemic. In Saudi Arabia, the number of cases of infection with COVID-19 continues to be markedly increasing over the last couple of weeks (2).

Given no specific proven antiviral therapy or vaccination available for COVID-19, the Nigella sativa (NS) seed oil is suggested as a potential supplement due to its known immunostimulant and antiviral activities in previous published studies (3,4). Fortunately, NS oil is available as an oral softgel capsules in commercial pharmacies in Saudi Arabia and licensed by the Saudi Food and Drug Authority (SFDA).

NS (known as Black seed) in Latin language or “Habatulbarakah” in Arabic language, is a well-known food supplement and medicinal plant in the Arabic and Islamic culture. It is used as a food spice and it has plenty of medical claims that originate from different historical backgrounds. The seeds of NS contain several active compounds that have been isolated, identified and reported, most importantly thymoquinone.

In the literature, NS showed several pharmacological activities including anti-inflammatory, anti-viral, and immunostimulatory activities (3,4).

## Overview of Non-Clinical Studies

Regarding antiviral activity, NS oil was found to suppress viral load of Murine cytomegalovirus (MCNV) in infected mice to undetectable level in the liver and spleen in 10 days’ intraperitoneal administration (5). This was possibly due to the increase in number and function of T cells and increased production of interferon-gamma. In addition, a review article discussed the potential antiviral activities of NS on different viruses including avian influenza (H9N2) in animal models. Interestingly, an in-vitro study showed the virus load decreased significantly when NS extract was added to the CoV infected cells and increased Interleukin-8 secretion (6).

Regarding immunostimulant activity, NS oil was found to exhibit nonspecific immunostimulant effect and able to induce cellular immune response as increasing lymphocytes pathogenesis and macrophage activities percent and macrophage index. It was found that it enhances T cells, natural killer cell activity and both interleukin-IB (IL-IB) and interleukine-3 (IL-3). Based on animal study, treatment with NS oil increased *the* phagocytic activity and phagocytic index of peritoneal macrophages and lymphocyte count in peripheral blood in diabetic hamsters (3). NS also reversed the Immunosuppression induced by lead and calcium in animal study. Based on laboratory study, a melanin extract from NS may induce the expression of three cytokines (TNF-alpha "Tumor necrosis factor alpha", IL-6 and VEGF "Vascular endothelial growth factor") that enhance immunogenicity (3).

Moreover, NS has also been shown to modulate the immune system by altering levels of inflammatory mediators (7). Also, due to other activities of NS such as anti-inflammatory, analgesic, antipyretic and bronchodilatation it may be beneficial to minimize the symptoms in COVID-19 patients (8,9,10).

## Overview of Clinical Studies

In a clinical study, 30 patients with hepatitis C virus (HCV) infection, who were not eligible for IFN- α/ribavirin therapy showed significant improvement in HCV viral load (16.67% became seronegative and 50% showing significant decrement) after NS course given over 3 months (4). In addition, according to a case report conducted by Onifade et al., after treatment with 10 mL of black seed twice daily for 6 months, a complete regaining and seroreversion of a 46-year-old HIV positive patient was evidenced (4).

***References***

*1. Lake MA. What we know so far: COVID-19 current clinical knowledge and research. Clin Med [Internet]. 2020 Mar 5 [cited 2020 Mar 27];20(2):124–7. Available from: http://www.ncbi.nlm.nih.gov/pubmed/32139372*

*2. Control TSC for DP and. Publications [Internet]. [cited 2020 Mar 27]. Available from:*

*https://covid19.cdc.gov.sa/professionals-health-workers/publications/*

*3. Ahmad A, Husain A, Mujeeb M, Khan SA, Najmi AK, Siddique NA, et al. A review on therapeutic potential of Nigella sativa: A miracle herb. Asian Pac J Trop Biomed [Internet]. 2013 May [cited 2013 Mar 20];3(5):337–52. Available from:*

*http://www.pubmedcentral.nih.gov/articlerender.fcgi?artid=3642442&tool=pmcentrez&rendertype=abstract*

*4. Molla S, Abul M, Azad K, Azam A, Hasib A, Monayem Hossain M, et al. A REVIEW ON ANTIVIRAL EFFECTS OF NIGELLA SATIVA L. pharmacologyonline [Internet]. 2019 [cited 2020 Mar 18];2:47–53.*

*Available from: http://pharmacologyonline.silae.it*

*5. Yimer EM, Tuem KB, Karim A, Ur-Rehman N, Anwar F. Nigella sativa L. (Black Cumin): A Promising Natural Remedy for Wide Range of Illnesses. Evid Based Complement Alternat Med [Internet]. 2019 [cited 2020 Mar 18];2019:1528635. Available from: http://www.ncbi.nlm.nih.gov/pubmed/31214267*

*6. Ulasli M, Gurses SA, Bayraktar R, Yumrutas O, Oztuzcu S, Igci M, et al. The effects of Nigella sativa* *(Ns), Anthemis hyalina (Ah) and Citrus sinensis (Cs) extracts on the replication of coronavirus and the expression of TRP genes family. Mol Biol Rep. 2014;41(3):1703–11.*

*7. Islam T. Nigellalogy: A Review on Nigella Sativa. MOJ Bioequivalence Bioavailab. 2017 Sep 25;3(6).*

*8. Koshak A, Wei L, Koshak E, Wali S, Alamoudi O, Demerdash A, et al. Immunomodulatory effects of Nigella sativa oil supplementation in patients with asthma: an RCT. In: The UCL Institute of Immunity and Transplantation Annual Immunology Symposium with Pears Lecture. Royal Free Campus, University College London, London, UK: The UCL Institute of Immunity and Transplantation; 2016.*

*9. Mady WH, Mady WH, Arafa A, Hussein AS, Aly MM, Madbouly HM. Nigella Sativa Oil as an Immunostimulant Adjuvant in H5 Based DNA Vaccine of H5N1 Avian Influenza Virus Based DNA Vaccine Highly Pathogenic Avian Influenza Virus H5N1 Nigella Sativa Oil Adjuvant Cell Mediated Immune Response Protective Efficacy Of H5 DNA Vaccine. Glob Vet. 2013;10(6):663–8.*

*10. Al-Ghamdi MS. The anti-inflammatory, analgesic and antipyretic activity of Nigella sativa. J Ethnopharmacol. 2001 Jun 1;76(1):45–8.*

*11. Gilani AH, Aziz N, Khurram IM, Chaudhary KS, Iqbal A. Bronchodilator, spasmolytic and calcium antagonist activities of Nigella sativa seeds (Kalonji): A traditional herbal product with multiple medicinal uses. J Pak Med Assoc [Internet]. 2001 Mar [cited 2016 Sep 11];51(3):115–20. Available from: http://jpma.org.pk/full_article_text.php?article_id=2562*

*12. Boskabady MH, Keyhanmanesh R, Khamneh S, Ebrahimi MA. The effect of Nigella sativa extract on tracheal responsiveness and lung inflammation in ovalbumin-sensitized guinea pigs. Clinics [Internet]. 2011 [cited 2014 May 28];66(5):879–87. Available from: http://www.scielo.br/scielo.php?script=sci_arttext&pid=S1807- 59322011000500027&lng=en&nrm=iso&tlng=en*

*13. Koshak A, Wei L, Koshak E, Wali S, Alamoudi O, Demerdash A, et al. Nigella sativa Supplementation Improves Asthma Control and Biomarkers: A Randomized, Double-Blind, Placebo-Controlled Trial. Phyther Res [Internet]. 2017 Jan 17 [cited 2017 Feb 1]; Available from: http://doi.wiley.com/10.1002/ptr.5761*

# STUDY RATIONALE

Today in the era of evidence-based medicine, it is hard to accept those traditional medical claims of medicinal plants without valid scientific experiments. Yet, it is important to rational the uses of these plants based on clinical evidence. Moreover, the world is currently facing a crisis because of this potentially fatal COVID-19 outbreak with no proven efficacy yet of any drug therapy, while vaccination is not expected until 2021. Therefore, this study is intended to evaluate the potential efficacy for using NS in patients with COVID-19

## Risk / Benefit Assessment

Potential benefits to individual participant: Anti-inflammatory and immunodulatory effects to enhance the recovery from COVID-19

Herbal product quality and safety: The Black Seed capsules (Cuminmar) are manufactured according to high standards of herbal product manufacturing facility and under GMP guidelines by Marnys Company. This product is licensed in Saudi Arabia under the local regulations (Saudi Food and Drug Authority) ensuring the quality and safety of drugs and sold in Saudi community pharmacies.

Investigators and Patient Safety: All procedures including patient diagnosis, primary and secondary endpoints measurements will be carried by specialist fully qualified medical doctors pharmacist inside King Abdulaziz university’s hospital.

# STUDY OBJECTIVES

## Primary Objective

The primary objective is to assess the clinical efficacy as measured by the change in clinical recovery period over the 10 days treatment period.

## Secondary Objectives

Assess the effect of NS on COVID-19 symptoms recovery.

# STUDY DESIGN

## Study Overview

This is a single center, open-label, controlled, randomized, phase II clinical trial. 200 of subjects are planned. Each subject will be administered a single dose (500 mg) of study drug twice daily for 10 days. Subjects will be assigned to the treatments in random order. Evaluations will be taken daily for evaulationg symptoms imrovpment.

Screening data will be reviewed to determine subject eligibility. Subjects who meet all inclusion criteria and none of the exclusion criteria will be entered into the study.

The following treatment regimens will be used:

- Experimental treatment NS at the following dose 500 mg twice daily
- Comparator – Standard care for COVID-19

Total duration of subject participation will be 14 days. Total duration of the study is expected to be 12 months.

# Criteria for evaluation

## Primary Efficacy Endpoint

Proportion of patients who clinically recovered (defined as 3 days of no symptoms) within 14 days after date of onset of symptoms.

## Secondary Efficacy Endpoints

- Improvement of Clinical Parameters:

o Time to full clinical recovery

o Time to progression to pneumonia or hypoxia

o Duration of illness

o Discharge to home

o Normalization of chest radiograph.

o Rate of complications: ICU admission, noninvasive ventilator, superadded infection

o Death.

- Normalization of Routine Laboratory Tests:

o Complete Blood Count (CBC)

o Erythrocyte sedimentation rate (ESR)

o C-reactive protein

o D-dimer

o Liver function test

o Renal function test

o Lactate Dehydrogenase (LDH),

o Immunoglobulins (IgA, IgE, IgG, IgM),

o Compliments (c3, c4),

o Chest X-ray, CT scan

o COVID-19 PCR

o Immune phenotype (CD3 T Cell, CD4 T Cell, CD8 T Cell, CD19 B Cell, CD20 B Cell, NK Cell)

- Additional:

o Inflammatory cytokines (IL-1β, IL-1Ra, IL-2, IL-6, IL-10, INF-alpha, INF-gamma, Eotaxin, RANTES & TNF-α).

## Safety Evaluations

Incidence of adverse events from the investigational drug or transfer to ICU

# SUBJECT SELECTION

## Study Population

Subjects with a diagnosis of mild to moderate COVID-19 who meet the inclusion and exclusion criteria will be eligible for participation in this study.

## Inclusion Criteria

-Patients with mild COVID19 (defined as upper respiratory tract infection symptoms in the absence of clinical or radiological signs of pneumonia).

-Adult (18 - 65 years old).

-Written informed consent by the patient (or legally authorized representative) prior to initiation of any study procedures.

-All patients should understand and agree to comply with planned study procedures.

-Polymerase chain reaction (PCR)-confirmed infection with Severe Acute Respiratory Syndrome Coronavirus-2 (SARS-CoV-2).

## Exclusion Criteria

-Patients with pneumonia or severe illness requiring admission to intensive care unit.

-Asymptomatic patients.

-Severe chronic kidney disease (i.e. estimated glomerular filtration rate [eGFR] < 30 mL/min) or end stage renal disease requiring dialysis

-Severe chronic liver disease (Alanine transaminase [AlT] or Aspartate transaminase [AST] > 5 times the upper limit of normal).

-Pregnancy or breast feeding.

-Anticipated transfer within 72 hours to another hospital that is not a study site.

-Allergy to the study medication.

# Concurrent Medications

All subjects should be maintained on the same medications throughout the entire study period, as medically feasible, with no introduction of new chronic therapies.

## Allowed Medications and Treatments

Standard therapy for COVID-19 is allowed except for treatments noted in the exclusion criteria described above and as noted in the prohibited medications section below. 

Prohibited Medications and Treatments: ANTICOAGULANT/ANTIPLATELET DRUGS, CNS DEPRESSANTS, CYCLOSPORINE, IMMUNOSUPPRESSANTS

# STUDY TREATMENTS

## Method of Assigning Subjects to Treatment Groups

Up to 200 eligible patients will be randomly assigned to treatment or control groups in a 1:1 ratio using a computer-generated randomization scheme developed by http://www.randomization.com.

## Blinding

NA

## Formulation of Test and Control Products

SFDA-licensed Marnys CUMINMAR *Nigella sativa* oil 500mg softgel oral capsules.

No control product will be used.

### Packaging and Labeling

*Packaging:* Study drug is supplied in cartons containing 3 strips of 20 single dose each. Each patient will receive 1 strip (20 single dose).

*Labeling example:* Each strip study drug will be labeled with the required SFDA statement, the protocol number, a treatment number, the name of the sponsors, and directions for patient use and storage.

## Supply of Study Drug at the Site

The Sponsor will ship Study Drug to the investigational sites. The initial study drug shipment will be shipped after site activation (i.e., all required regulatory documentation has been received by the Sponsor and a contract has been executed). Subsequent study drug shipments will be made after site request for resupply.

### Dosage/Dosage Regimen

*Nigella sativa* oil 500mg softgel capsules in oral twice daily dose for 10 days

### Dispensing

The investigator will dispense the drug at the participation site

### Administration Instructions

Take 1 capsule with plenty glass of water.

## Supply of Study Drug at the Site

the study drug will be delivered from the supplier to the investigational site.

### Storage

Study drug should be stored by the study site at controlled room temperature, 15 to 25ºC in a dry place away from light. If the temperature of study drug storage in the clinic/pharmacy exceeds or falls below this range, this should be reported to the Sponsor or designee and captured as a deviation. Subjects will be instructed to store the medication in original packaging at room temperature according to the instructions outlined on the Drug Administration Instructions.

## Study Drug Accountability

An accurate and current accounting of the dispensing and return of study drug for each subject will be maintained on an ongoing basis by the investigator.

## Measures of Treatment Compliance

Subjects will be monitored on daily bases during the study period for their study drug intake and any adverse events.

# STUDY PROCEDURES AND GUIDELINES

A Schedule of Events representing the required testing procedures to be performed for the duration of the study is diagrammed in Appendix 1.

Prior to conducting any study-related activities, informed consent will be taken by the subject or subject’s legal representative. If appropriate, assent must also be obtained prior to conducting any study-related activities.

## Clinical Assessments

### Concomitant Medications

All concomitant medication and concurrent therapies will be documented at Baseline/Screening and at Study Days and at early termination when applicable. Dose, route, unit frequency of administration, and indication for administration and dates of medication will be captured.

### Demographics

Demographic information (date of birth, gender, race) will be recorded at Screening.

### Medical History

Relevant medical history, including history of current disease, other pertinent respiratory history, and information regarding underlying diseases will be recorded at Screening.

### Physical Examination

A complete physical examination will be performed by either the physician of the research team at study site. Qualified staff (MD, NP, RN, and PA) may complete the abbreviated physical exam at all other visits. New abnormal physical exam findings must be documented and will be followed by a physician or other qualified staff.

### Adverse Events

Information regarding occurrence of adverse events will be captured throughout the study. Duration (start and stop dates and times), severity/grade, outcome, treatment and relation to study drug will be recorded on the case report form (CRF).

## Clinical Laboratory Measurements

NA

# EVALUATIONS BY VISIT

## Visit 1 (Day 1)

1. Review the study with the subject (subject’s legal representative) and obtain written informed consent.
2. Assign the subject a unique screening number.
3. Record demographics data.
4. Record medical history, diagnosis date.
5. Record concomitant medications.
6. Randomize subject,
7. Dispense study drug,
8. Initiate subject diary in CRF.

## 14 days monitoring

1. Record patients’ symptoms
2. Record any Adverse Experiences and dosing compliance.
3. Concomitant medications review.

# ADVERSE Experience REPORTING AND DOCUMENTATION

## Adverse Events

An adverse event (AE) is any untoward medical occurrence in a clinical investigation of a patient administered a pharmaceutical product and that does not necessarily have a causal relationship with the treatment. An AE is therefore any unfavorable and unintended sign (including an abnormal laboratory finding), symptom or disease temporally associated with the administration of an investigational product, whether or not related to that investigational product. An unexpected AE is one of a type not identified in nature, severity, or frequency in the current Investigator’s Brochure or of greater severity or frequency than expected based on the information in the Investigator’s Brochure.

The Investigator will probe, via discussion with the subject, for the occurrence of AEs during each subject visit and record the information in the site’s source documents. Adverse events will be recorded in the patient CRF. Adverse events will be described by duration (start and stop dates and times), severity, outcome, treatment and relation to study drug, or if unrelated, the cause.

**AE Severity**

The National Cancer Institute’s Common Terminology Criteria for Adverse Events (CTCAE) Version 3.0 should be used to assess and grade AE severity, including laboratory abnormalities judged to be clinically significant. The modified criteria can be found in the study manual. If the experience is not covered in the modified criteria, the guidelines shown in Table 1 below should be used to grade severity. It should be pointed out that the term “severe” is a measure of intensity and that a severe AE is not necessarily serious.

Table 1. AE Severity Grading

| **Severity (Toxicity Grade)** | **Description** |
| --- | --- |
| Mild (1) | Transient or mild discomfort; no limitation in activity; no medical intervention or therapy required. The subject may be aware of the sign or symptom but tolerates it reasonably well. |
| Moderate (2) | Mild to moderate limitation in activity, no or minimal medical intervention/therapy required. |
| Severe (3) | Marked limitation in activity, medical intervention/therapy required, hospitalizations possible. |
| Life-threatening (4) | The subject is at risk of death due to the adverse experience as it occurred. This does not refer to an experience that hypothetically might have caused death if it were more severe. |

**AE Relationship to Study Drug**

The relationship of an AE to the study drug should be assessed using the following the guidelines in Table 2.

Table 2. AE Relationship to Study Drug

| **Relationship to Drug** | **Comment** |
| --- | --- |
| Definitely | Previously known toxicity of agent; or an event that follows a reasonable temporal sequence from administration of the drug; that follows a known or expected response pattern to the suspected drug; that is confirmed by stopping or reducing the dosage of the drug; and that is not explained by any other reasonable hypothesis. |
| Probably | An event that follows a reasonable temporal sequence from administration of the drug; that follows a known or expected response pattern to the suspected drug; that is confirmed by stopping or reducing the dosage of the drug; and that is unlikely to be explained by the known characteristics of the subject’s clinical state or by other interventions. |
| Possibly | An event that follows a reasonable temporal sequence from administration of the drug; that follows a known or expected response pattern to that suspected drug; but that could readily have been produced by a number of other factors. |
| Unrelated | An event that can be determined with certainty to have no relationship to the study drug. |

## Serious Adverse Experiences (SAE)

An SAE is defined as any AE occurring at any dose that results in any of the following outcomes:

- death
- a life-threatening adverse experience
- inpatient hospitalization or prolongation of existing hospitalization
- a persistent or significant disability/incapacity
- a congenital anomaly/birth defect

Other important medical events may also be considered an SAE when, based on appropriate medical judgment, they jeopardize the subject or require intervention to prevent one of the outcomes listed.

### Serious Adverse Experience Reporting

Study sites will document all SAEs that occur (whether or not related to study drug) per [SFDA Guidelines](http://www.research.ucsf.edu/chr/Guide/Adverse_Events_Guidelines.asp#2). The collection period for all SAEs will begin after informed consent is obtained and end after procedures for the final study visit have been completed.

In accordance with the standard operating procedures and policies of the local Institutional Review Board (IRB)/Independent Ethics Committee (IEC), the site investigator will report SAEs to the IRB/IEC.

## Medical Monitoring

Dr Abdulrahman Koshak should be contacted directly at these numbers to report medical concerns or questions regarding safety.

Phone: +966 543292333

# DISCONTINUATION And Replacement of subjects

## Early Discontinuation of Study Drug

A subject may be discontinued from study treatment at any time if the subject, the investigator, or the Sponsor feels that it is not in the subject’s best interest to continue. The following is a list of possible reasons for study treatment discontinuation:

- Subject withdrawal of consent (or assent)
- Subject is not compliant with study procedures
- Adverse event that in the opinion of the investigator would be in the best interest of the subject to discontinue study treatment
- Protocol violation requiring discontinuation of study treatment
- Lost to follow-up
- Sponsor request for early termination of study
- Positive pregnancy test (females)

If a subject is withdrawn from treatment due to an adverse event, the subject will be followed and treated by the medical research team until the abnormal parameter or symptom has resolved or stabilized.

All subjects who discontinue study treatment should come in for an early discontinuation visit as soon as possible and then should be encouraged to complete all remaining scheduled visits and procedures.

All subjects are free to withdraw from participation at any time, for any reason, specified or unspecified, and without prejudice.

Reasonable attempts will be made by the investigator to provide a reason for subject withdrawals. The reason for the subject’s withdrawal from the study will be specified in the subject’s source documents Refer to Section 10 for early termination procedures.

## Withdrawal of Subjects from the Study

A subject may be withdrawn from the study at any time if the subject, the investigator, or the Sponsor feels that it is not in the subject’s best interest to continue.

All subjects are free to withdraw from participation at any time, for any reason, specified or unspecified, and without prejudice.

Reasonable attempts will be made by the investigator to provide a reason for subject withdrawals.  The reason for the subject’s withdrawal from the study will be specified in the subject’s source documents.  As noted above, subjects who discontinue study treatment early (i.e., they withdraw prior to Visit 1) should have an early discontinuation visit. Refer to Section 10 for early termination procedures.  Subjects who withdraw after Visit 1 should be encouraged to come in for a final visit (and the procedures to be followed would include those for their next scheduled visit).

## Replacement of Subjects

Subjects who withdraw from the study treatment will be replaced.

# PRotocol Violations

A protocol violation occurs when the subject, investigator, fails to adhere to significant protocol requirements affecting the inclusion, exclusion, subject safety and primary endpoint criteria. Protocol violations for this study include, but are not limited to, the following:

- Failure to meet inclusion/exclusion criteria
- Use of a prohibited concomitant medication

Failure to comply with Good Clinical Practice (GCP) guidelines will also result in a protocol violation. The Sponsor will determine if a protocol violation will result in withdrawal of a subject.

When a protocol violation occurs, it will be discussed with the investigator and a Protocol Violation Form detailing the violation will be generated. This form will be signed by a Sponsor representative and the Investigator. A copy of the form will be filed in the site’s regulatory binder and in the Sponsor’s files.

# STATISTICAL METHODS AND CONSIDERATIONS

## Data Sets Analyzed

All dosed subjects will be included in data analysis

## Demographic and Baseline Characteristics

The following demographic variables at screening will be summarized by dose level: gender, age, height and weight.

## Analysis of Primary Endpoint

*Student t test will be used for significance analysis.*

## Analysis of Secondary Endpoints

*Student t test will be used for significance analysis.*

## Sample Size and Randomization

To achieve a power of 80% and a two-tailed p-value of <0.05 of an assumed 40% difference between the proportion of patients with clinical cure among the NS group in comparison with the control group, a total of at least 200 patients assigned randomly into the two arms (ie 100 patients per arm) will be needed to be included in the study as calculated using G*Power version 3.1.9.2 (Heinrich-Heine-University Düsseldorf, Düsseldorf, Germany).

# DATA COLLECTION, RETENTION AND MONITORING

## Data Collection Instruments

The Investigator will prepare and maintain adequate and accurate source documents designed to record all observations and other pertinent data for each subject treated with the study drug.

Study personnel at each site will enter data from source documents corresponding to a subject’s visit into the protocol-specific paper CRF when the information corresponding to that visit is available. Subjects will not be identified by name in the study database or on any study documents to be collected by the Sponsor (or designee), but will be identified by a subject number.

The Investigator is responsible for all information collected on subjects enrolled in this study. All data collected during the course of this study must be reviewed and verified for completeness and accuracy by the Investigator. A copy of the CRF will remain at the Investigator’s site at the completion of the study.

## Data Management Procedures

The data will be entered into a validated database. The investigator will be responsible for data processing, in accordance with procedural documentation.

All procedures for the handling and analysis of data will be conducted using good computing practices meeting SFDA guidelines for the handling and analysis of data for clinical trials.

## Data Quality Control and Reporting

NA

## Archival of Data

The database is safeguarded against unauthorized access by established security procedures; appropriate backup copies of the database and related software files will be maintained.  Databases are backed up by the database administrator in conjunction with any updates or changes to the database.

At critical junctures of the protocol (e.g., production of interim reports and final reports), data for analysis is locked and cleaned per established procedures.

## Availability and Retention of Investigational Records

The Investigator must make study data accessible to the monitor, other authorized representatives of the Sponsor (or designee), IRB/IEC, and Regulatory Agency (e.g., SFDA) inspectors upon request. A file for each subject must be maintained that includes the CRF. The Investigator must ensure the reliability and availability of source documents from which the information on the CRF was derived.

All study documents (copies of CRFs) must be kept secured for a period of two years following marketing of the investigational product or for two years after centers have been notified that the IND has been discontinued. There may be other circumstances for which the Sponsor is required to maintain study records and, therefore, the Sponsor should be contacted prior to removing study records for any reason.

## Monitoring

NA

## Subject Confidentiality

In order to maintain subject confidentiality, only a site number, subject number and subject initials will identify all study subjects on CRFs and other documentation submitted to the Sponsor. Additional subject confidentiality issues (if applicable) are covered in the Clinical Study Agreement.

# ADMINISTRATIVE, ETHICAL, REGULATORY CONSIDERATIONS

The study will be conducted according to the Declaration of Helsinki, Protection of Human Volunteers (21 CFR 50), Institutional Review Boards (21 CFR 56), and Obligations of Clinical Investigators (21 CFR 312).

To maintain confidentiality, all laboratory specimens, evaluation forms, reports and other records will be identified by a coded number and initials only. All study records will be kept in a locked file cabinet and code sheets linking a patient’s name to a patient identification number will be stored separately in another locked file cabinet. Clinical information will not be released without written permission of the subject, except as necessary for monitoring by the SFDA. The Investigator must also comply with all applicable privacy regulations (e.g., Health Insurance Portability and Accountability Act of 1996, EU Data Protection Directive 95/46/EC).

## Protocol Amendments

Any amendment to the protocol will be written by the Sponsor. Protocol amendments cannot be implemented without prior written IRB/IEC approval except as necessary to eliminate immediate safety hazards to patients. A protocol amendment intended to eliminate an apparent immediate hazard to patients may be implemented immediately, provided the IRBs are notified within five working days.

## Institutional Review Boards and Independent Ethics Committees

The protocol and consent form will be reviewed and approved by the IRB/IEC of King Abdulaziz University Hospital. Serious adverse experiences regardless of causality will be reported to the IRB/IEC in accordance with the standard operating procedures and policies of the IRB/IEC, and the Investigator will keep the IRB/IEC informed as to the progress of the study. The Investigator will obtain assurance of IRB/IEC compliance with regulations.

Any documents that the IRB/IEC may need to fulfill its responsibilities (such as protocol, protocol amendments, Investigator’s Brochure, consent forms, information concerning patient recruitment, payment or compensation procedures, or other pertinent information) will be submitted to the IRB/IEC. The IRB/IECs written unconditional approval of the study protocol and the informed consent form will be in the possession of the Investigator before the study is initiated. This approval must refer to the study by exact protocol title and number and should identify the documents reviewed and the date of review.

Protocol and/or informed consent modifications or changes may not be initiated without prior written IRB/IEC approval except when necessary to eliminate immediate hazards to the patients or when the change(s) involves only logistical or administrative aspects of the study. Such modifications will be submitted to the IRB/IEC and written verification that the modification was submitted and subsequently approved should be obtained.

The IRB/IEC must be informed of revisions to other documents originally submitted for review; serious and/or unexpected adverse experiences occurring during the study in accordance with the standard operating procedures and policies of the IRB; new information that may affect adversely the safety of the patients of the conduct of the study; an annual update and/or request for re-approval; and when the study has been completed.

## Informed Consent Form

Informed consent will be obtained in accordance with the Declaration of Helsinki, ICH GCP, US Code of Federal Regulations for Protection of Human Subjects (21 CFR 50.25[a,b], CFR 50.27, and CFR Part 56, Subpart A), the Health Insurance Portability and Accountability Act (HIPAA, if applicable), and local regulations.

The Investigator will prepare the informed consent form and submit to the IRB/IEC. The consent form generated by the Investigator must be acceptable to the Sponsor and be approved by the IRB/IEC. The written consent document will embody the elements of informed consent as described in the International Conference on Harmonisation and will also comply with local regulations. The Investigator will send an IRB/IEC-approved copy of the Informed Consent Form to the Sponsor (or designee) for the study file.

A properly executed, verbal informed consent will be obtained from each subject prior to entering the subject into the trial. Information should be given in both oral and written form and subjects (or their legal representatives) must be given ample opportunity to inquire about details of the study. If appropriate and required by the local IRB/IEC, assent from the subject will also be obtained.

## Publications

The preparation and submittal for publication of manuscripts containing the study results shall be in accordance with a process determined by mutual written agreement among the study Sponsor and participating institutions. The publication or presentation of any study results shall comply with all applicable privacy laws, including, but not limited to, the Health Insurance Portability and Accountability Act of 1996.

## Investigator Responsibilities

By signing the Agreement of Investigator form, the Investigator agrees to:

1. Conduct the study in accordance with the protocol and only make changes after notifying the Sponsor (or designee), except when to protect the safety, rights or welfare of subjects.
2. Personally, conduct or supervise the study (or investigation).
3. Ensure that the requirements relating to obtaining informed consent and IRB review and approval meet federal guidelines, as stated in § 21 CFR, parts 50 and 56.
4. Report to the Sponsor or designee any AEs that occur in the course of the study, in accordance with §21 CFR 312.64.
5. Ensure that all associates, colleagues and employees assisting in the conduct of the study are informed about their obligations in meeting the above commitments.
6. Maintain adequate and accurate records in accordance with §21 CFR 312.62 and to make those records available for inspection with the Sponsor (or designee).
7. Ensure that an IRB that complies with the requirements of §21 CFR part 56 will be responsible for initial and continuing review and approval of the clinical study.
8. Promptly report to the IRB and the Sponsor (or designee) all changes in the research activity and all unanticipated problems involving risks to subjects or others (to include amendments and IND safety reports).
9. Seek IRB approval before any changes are made in the research study, except when necessary to eliminate hazards to the patients/subjects.
10. Comply with all other requirements regarding the obligations of clinical investigators and all other pertinent requirements listed in § 21 CFR part 312
